# HPMCD: the database of human microbial communities from metagenomic datasets and microbial reference genomes

**DOI:** 10.1093/nar/gkv1216

**Published:** 2015-11-17

**Authors:** Samuel C. Forster, Hilary P. Browne, Nitin Kumar, Martin Hunt, Hubert Denise, Alex Mitchell, Robert D. Finn, Trevor D. Lawley

**Affiliations:** 1 Host Microbiota Interactions Laboratory, Wellcome Trust Sanger Institute, Wellcome Genome Campus, Hinxton CB10 1SA, UK; 2 Centre for Innate Immunity and Infectious Diseases, Hudson Institute of Medical Research, Clayton 3168, Australia; 3 Department of Molecular and Translational Sciences, Monash University, Clayton 3800, Australia; 4 Pathogen Informatics, Wellcome Trust Sanger Institute, Wellcome Genome Campus, Hinxton CB10 1SA, UK; 5 European Molecular Biology Laboratory, European Bioinformatics Institute, Wellcome Genome Campus, Hinxton CB10 1SD, UK

## Abstract

The Human Pan-Microbe Communities (HPMC) database (http://www.hpmcd.org/) provides a manually curated, searchable, metagenomic resource to facilitate investigation of human gastrointestinal microbiota. Over the past decade, the application of metagenome sequencing to elucidate the microbial composition and functional capacity present in the human microbiome has revolutionized many concepts in our basic biology. When sufficient high quality reference genomes are available, whole genome metagenomic sequencing can provide direct biological insights and high-resolution classification. The HPMC database provides species level, standardized phylogenetic classification of over 1800 human gastrointestinal metagenomic samples. This is achieved by combining a manually curated list of bacterial genomes from human faecal samples with over 21000 additional reference genomes representing bacteria, viruses, archaea and fungi with manually curated species classification and enhanced sample metadata annotation. A user-friendly, web-based interface provides the ability to search for (i) microbial groups associated with health or disease state, (ii) health or disease states and community structure associated with a microbial group, (iii) the enrichment of a microbial gene or sequence and (iv) enrichment of a functional annotation. The HPMC database enables detailed analysis of human microbial communities and supports research from basic microbiology and immunology to therapeutic development in human health and disease.

## INTRODUCTION

The importance of microbial communities and the complex functional roles they perform in human health and disease is becoming increasingly evident (1–4). Large-scale projects such as the Human Microbiome Project and MetaHIT, have provided considerable advancements in this area of research (1,2). The growing availability of metagenomic sequencing and associated analysis tools has enabled quantification and understanding of this microbial diversity at a level not previously possible. While traditional 16S rRNA gene based profiling approaches were limited to bacteria and archaea, whole genome sequence or shotgun metagenomics, expands analysis to eukaryotic, fungal and DNA viruses that do not possess a 16S rRNA gene. Coupled with computational analysis and high quality reference genomes this enables higher phylogenetic resolution (e.g. species or even strain level relationships), where no differences would be detectable using only 16S rRNA gene profiling.

The importance of understanding the tremendous molecular and phenotypic diversity present within single bacterial ‘species’ or lineage has only recently become evident. Whole genome phylogeny of hundreds of isolates from a single ‘species’ has demonstrated each ‘species’ represents an evolutionary web of highly related lineages with diverse phenotypic characteristics (5–8). For example, strains of many opportunistic pathogen that cannot be differentiated by 16S rRNA gene profiling such as *Peptoclostridium difficile* or *Escherichia coli*, can range from benign members of the gastrointestinal tract to highly virulent pathogens, inducing severe, sometimes fatal symptoms in the host (5,9,10). Though less well studied, similarly important phylogenetic relationships are also likely to occur in fungal and viral species.

Although many excellent repositories for metagenomic sequence datasets already exist, these tools serve primarily to support wide-scale submission, data archive and generic analysis functions suitable for microbiota across many environments (11–13), are focused on microbial function rather than community structure (11,14) or are limited in scope to specific studies or datasets (15) (Supplementary Table S1). In the human gastrointestinal tract, focused research effort has resulted in considerable sampling, underlying biological knowledge and high quality reference genomes to enable specialized analysis approaches. The microbiota of the human gastrointestinal tract is estimated to include between 500 and 1000 species, and plays a critical role in our sustenance, immune system development and protection against infection (1). In this important area of research, the ability to incorporate a comprehensive measure of microbial diversity with high phylogenetic resolution will facilitate the progression from current basic, correlation-based observational studies to microbe identification and experimental validation of causative relationships. In human health, this capacity will provide insights from basic biology and disease understanding to identification of biologically relevant biomarkers and ultimately inform targeted therapeutic intervention (16,17).

We present the Human Pan-Microbial Communities (HPMC) database, a database of human gastrointestinal microbiota derived from faecal samples. The HPMC database (v1.15.5) currently incorporates 1830 independent samples including 4425 whole genome metagenomics sequencing runs available in the European Nucleotide Archive (18). The human faecal derived samples correspond to approximately 41% of all public EBI-metagenomics portal samples (12) and 29% of all MG-RAST public whole genome metagenomic samples (19). These samples were subjected to stringent quality control and a standardized, specifically designed analysis process described in detail below. This process leverages an optimized, manually curated list of over 21 000 microbial genomes for sequence read classification. Classification in this manner provides species and the potential for strain level resolution and also includes other important viral, fungal and eukaryotic members of the microbiota community that are undetectable with 16S rRNA gene profiling. In addition, the raw metagenomic sequences are utilized to support extensive functional analysis and sequence-based search functionality. This enhanced classification and comprehensive sequence analysis is supplemented with manually curated metadata to facilitate complex, multidimensional sample filtering and search queries across currently disparate datasets at the sample, species, functional and sequence level.

## DATA SOURCES AND PROCESSING

The HPMC database represents a highly specialized, human sample specific extension to the standard EBI metagenomics portal (EMP). Sample data are integrated from high quality metagenomics datasets within the EMP that are enhanced with manually curated sample metadata and a culture derived, comprehensive genome collection for phylogenetic analysis. Samples are considered for inclusion in the database where they originate from human faecal samples and contain sufficient metadata to determine if they originate from a healthy of diseased individual. Samples where >25% of reads are filtered due to poor read quality or significant human contamination are excluded at this point. In the current version of the database 94 samples were excluded by this filtering. Historical, publicly available metagenomic samples that are available in the European Nucleotide Archives but are currently absent in the EMP and pass these quality criteria have also been included. Reads from included samples undergo quality filtering with Trimommatic v0.33 (20), high quality gene fragments are identified using FragGeneScan v1.19 (21) and functional annotation performed using InterProScan v5.0 (22) as described previously (12). Identified gene fragments are also included in a reference BLAT database to provide sequence similarity search functionality (23).

One of the fundamental features of the HPMC database lies in the ability to provide detailed taxonomic resolution for gastrointestinal microbes. The Kraken algorithm (24) provides the ability to classify whole genome metagenomic reads based on a *k*-mer lowest common ancestor database generated from whole genome sequences. The HPMC analysis process applies the Kraken v0.10.6 approach to classify reads against a custom database populated with complete bacterial, archaeal, fungal and viral genomes including 216 bacterial genomes derived from bacterial cultures isolated directly from human faecal samples.

Knowledge of the gastrointestinal tract specifically is incorporated within the HPMC to generate a ‘gold-standard,’ manually curated list of species that have been reported as experimentally cultured from faecal samples. In addition, it is also possible for users to register for a free account on HPMC database. Registration enables users to independently annotate species as known members of the gastrointestinal microbiota. These annotations are saved to the users personal account and contribute to the overall, community-wide, classification of gastrointestinal species available for searching and filtering by all users of the HPMC database.

**Figure 1.**
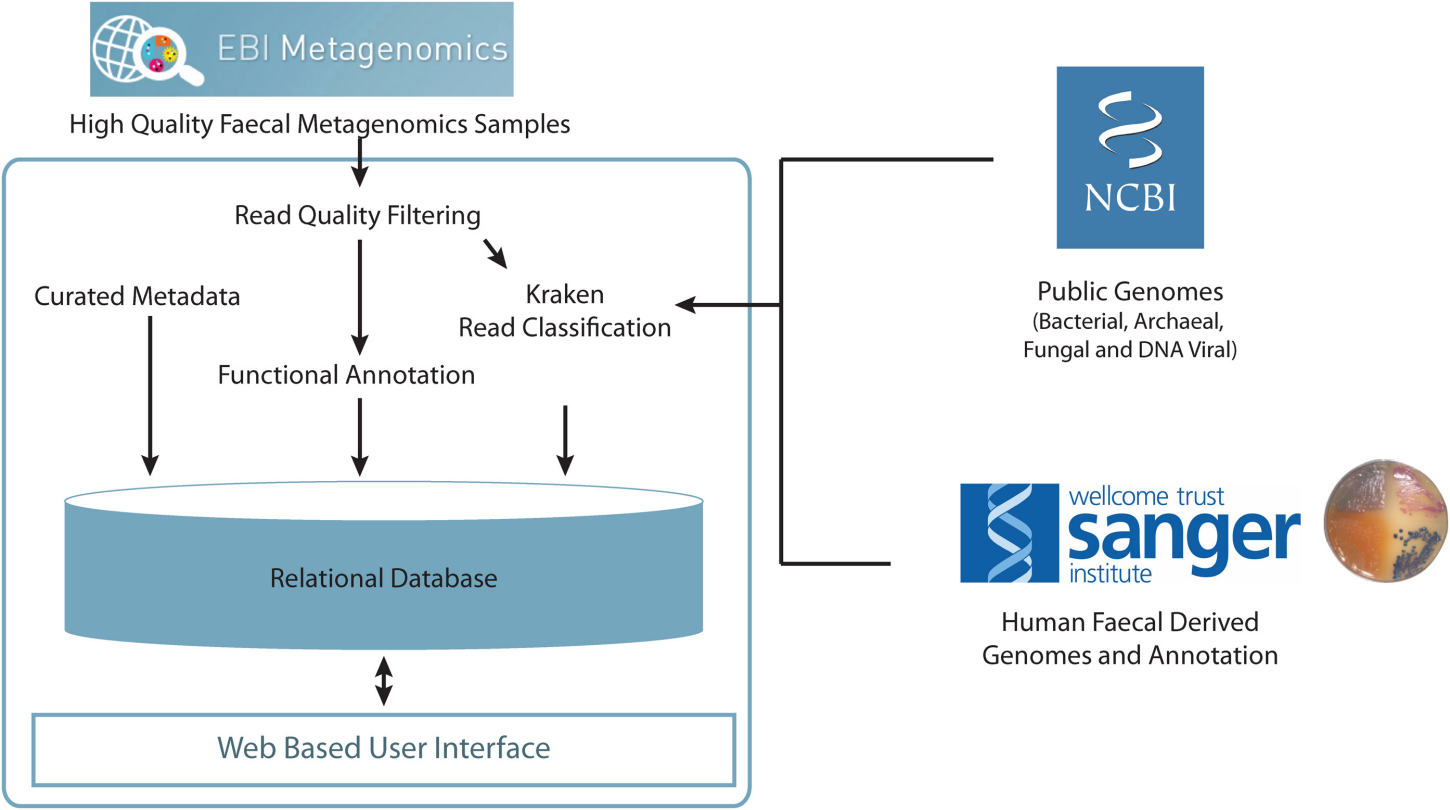
Overview of HPMC database structure and analysis process. High quality sequences from human faecal metagenomic samples are combined with manually curated and sample metadata. Phylogenetic classification is performed in the context of over 21000 curated genomes. Sample and functional annotation data are stored in a relational database and available for querying through a freely available web based interface.

Quantification and comparison of metagenomic samples is dependent on accurate normalization between phylogenetic groups, samples and experiments. Variability in the availability of genomes from different phylogenetic groups impacts the classification potential when applying the lowest common ancestor approach. For example, while classification to the species level when only a few representative species are described within a genus can be readily achieved, accurate species classification is unlikely due to poor representation across species diversity. Inclusion of many species, such as employed in the HPMC database increases the percentage of reads that are only able to be classified at the genus level due to extensive gene homology. It is therefore necessary to correct for this bias prior to performing conventional transformations, standardization and sample scaling. The HPMC overcomes this limitation by correcting assigned read counts at the phylogenetic level by genome uniqueness. Genome uniqueness is defined as the percentage of the genome where a 100-bp sliding window would uniquely identify that genome amongst all genomes contained in the database. This approach corrects for uneven genome coverage across phylogenetic group enabling direct comparison between species. The resulting corrected counts are subjected to log transformation, samples standardized to a mean of 0 and multiple sample scaling performed according to best practice metagenomic data analysis (19). This approach provides the ability to perform comprehensive metagenomic analysis across diverse phylogenetic groups with variable genome representation (Figure 1).

To facilitate complex, multi-faceted search functionality and supplement incomplete and poorly annotated metagenomics samples, manual searching of the original published articles were performed for each study included in the HPMC database to supplement metadata and capture the maximum information available for each sample. This approach supplements the minimal data required for submission to public repositories and updates metadata associated with older sample submissions to reflect current standards. The results of metagenomic sample analysis and functional annotation were combined with this manually curated metadata into a relational database designed to support complex querying and advanced analysis functionality.

## ANALYSIS CAPABILITY

**Figure 2.**
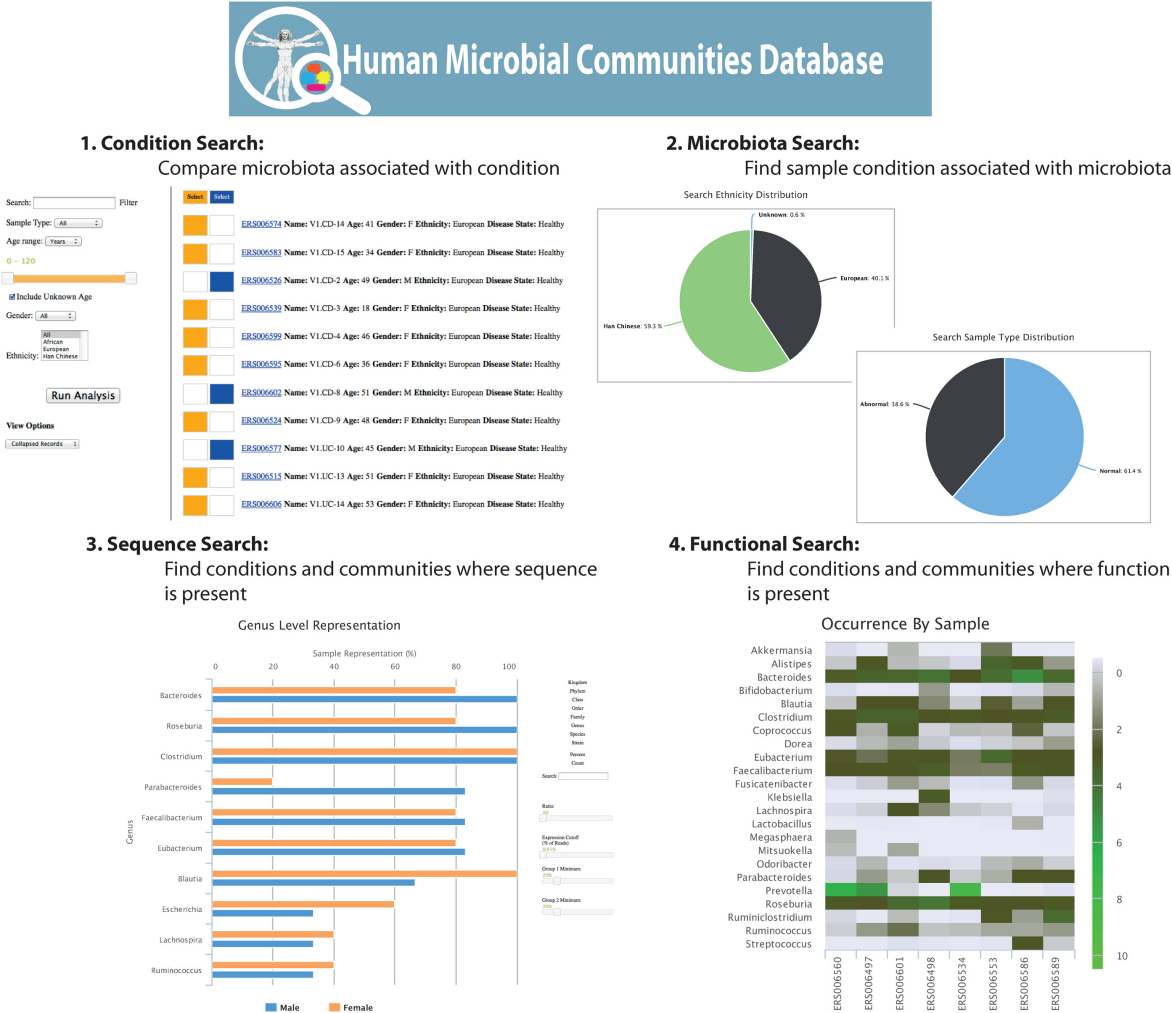
Condition and Microbiota Search Functionality. The search functionality provides the ability to query by (1) samples (filtered by manually curated metadata), (2) specific microbes, (3) sequence similarity or (4) functional annotation. Summary data about the identified samples and microbiota composition are provided as raw data, bar charts, heat maps and pie charts.

The HPMC database is designed to support 4 types of searches. These are to: (i) search by sample facet (i.e. human health or disease state) to identify microbiota compositions or specific microbial groups, (ii) search by microbiota composition or specific microbial groups to identify sample type and community correlations, (iii) search by sequence (i.e. microbiota or other gene sequence) or (iv) search by functional annotation to identify associated sample type and microbial groups (Figure 2).

### Search for microbial groups associated with health or disease state

The sample search function is designed for users with knowledge of the host biology and interest in the microbiota that correlates with health or disease state. The search interface allows the user to perform detailed metadata based sample filtering by parameters including health status, age, gender, geography and ethnicity. Samples can be allocated to one of two user-defined groups with comparisons performed between the microbiota communities in each condition. For example, one may wish to compare the microbiota detected in samples with a particular disease state such as Inflammatory Bowel Disease (IBD) with all other healthy samples in the database. On performing this analysis a clear decrease is observed in the representation of Firmicutes and Bacteroidetes in the community associated with the disease state. At the species level, analysis suggests a loss of *Faecalibacterium prausnitzii*, *Bacteroides vulgatus* and two species of the Roseburia genus (*R. hominis* and *R. faecis*). This result is consistent with the core EMP that suggests a decrease in one or more of the 193 known Ruminococcaceae and one or more of the 713 known Lachnospiraceae family members. These results demonstrate the improved resolutions provided by the HPMC with the loss of both *F. prausnitzii* and Roseburia species previously reported to be associated with IBD (25–27). Functionality is also provided to further filter this search to include only samples obtained from a particular ethnicity, gender or age profile. The user interface provides full flexibility to this search approach, supporting the comparison of any two groups of samples filtered by any combination of search parameters.

### Search for health or disease states associated with presence of microbial group

While the majority of existing metagenomic analysis focuses on identification of the microbial compositions within particular samples, the HPMC database also provides the ability to search by a component of the microbiota. For example, one may be interested to identify the sample conditions and microbial community structure where *Helicobacter pylori*, a common risk factor for gastric cancer, exists in healthy individuals. The search functionality provided by the HPMC database enables identification of the samples where a microbial component or phylogenetic group, such as *H. pylori*, is represented at a level higher or lower than is observed across the complete population of samples within the database. This analysis is particularly powerful for researchers with expertise in specific microbes and provides the ability to identify previously unknown conditions in which the microbes are dominant. In addition to the analysis of the sample condition in which the microbial group of interest occurs, the HPMC database also enables users to analyse the associated community structure. This analysis enables the identification of all groups, at any phylogenetic level, that regularly co-occur, or never co-occur with the species of interest. This is achieved by comparing those samples in which the microbial group of interest was detected to the background composition of all datasets. Analysis of co-occurring species and community structure can provide insights into health associated microbiota communities and assist in the prediction of host interactions and responses.

### Search by enrichment of a microbial gene or sequence

Expanding on the benefits of whole genome metagenomic sequencing over traditional 16S rRNA gene profiling, the HPMC database provides the ability to search for microbial genes or sequences of interest directly detected within the sample. Each sample is characterized by the presence, defined as detection above a user-defined percentage similarity, of a particular searched sequence. The samples in which the searched sequence is detected are compared to the remaining samples within the database where the sequence was not found to be present at the defined homology cut-off. The sequence search functionality enables users to search independently of known, curated functional information. For example, if one discovers a novel antibiotic resistance gene, the HPMC database provides the ability to determine the conditions in which species possessing this gene are detected and identify common microbiota community structures and sample conditions associated with this gene of interest.

### Search by enrichment of a functional annotation

To complement the sequence homology searching, the HPMC database also provides the ability to search for samples with specific functional annotations. This search functionality enables detailed identification of sample types or community structures typically associated with a particular biological function. As described for sequence homology based searches, samples will be compared based on presence or absence of the defined functional annotation. This approach enables the discovery of sample conditions and microbiota community structure specific to the functional annotation, such as virulence, metabolism, sporulation and immune system evasion. Knowledge of the conditions where functional capacity is present or absent is fundamental to the development of the basic biological understanding needed to further microbiota research.

## DATA AVAILABILITY AND SUPPORT

To ensure compatibility with existing resources, standardized ERS/SRS identifiers are used to identify raw reads and NCBI identifiers are used for genome identification. Direct links are provided to the relevant records on these sites where appropriate. Analysis results are also provided for export as a single download file to support further investigation. Throughout all search results, images are available for export in PDF, PNG, JPG and SVG formats. Complete raw data for the entire database is available as a flat-file download from the help pages (http://www.hpmcd.org/help.php).

## FUTURE DEVELOPMENTS

The close association between the HPMC database and the EMP ensures continued inclusion of newly published, relevant human metagenomic studies as they become publicly available. This important linkage ensures that the HPMC database will continue to provide access to the most comprehensive selection of human gastrointestinal metagenomic datasets complemented by a specialized analysis process and detailed manual curation.

The sophisticated analysis framework will continue to be expanded, providing additional search functionality consistent with the standard metadata requirements incorporated in the data submission process. Improvements in the complexity and specificity of search functionality will also become possible as the diversity of samples increases to include further, diverse infections, disease conditions and sample types.

As the user base continues to expand the comprehensive nature of the list of cultured species and community annotated gastrointestinal species annotation will also increase. Over time this resource will grow to become a unique, community consensus of human gastrointestinal bacteria, providing an extra level of annotation for analysis of metagenomic datasets. In parallel, as the diversity of complete reference genomes from cultured species expands and the number of whole genome metagenomic experiments increases, the scope of the HPMC database will also be widened from the current focus on human gastrointestinal tract to encompass samples from other human body sites including female reproductive tract, lungs and bladder within the same computational framework.

## CONCLUSIONS

The HPMC expands on the general-purpose metagenomic analysis capabilities provided by the EMP to provide a tailored analysis platform capable of supporting the specific, search requirements for human metagenomic data and medical research. As the use of whole genome metagenomic sequencing expands and the number of high quality reference genomes increases, the ability to perform integrated analysis of samples from multiple independent studies becomes increasingly necessary. While many individual studies may suffer from limited statistical power due to small sample size, integrated meta-analysis as provided in the HPMC database overcomes many of these limitations. In this context, the HPMC database represents the next step in metagenomic analysis, supporting the progression from generic sample archive and correlation studies to biologically relevant candidate identification suitable for direct experimental validation and human medical applications.
